# KISS—Keep It Static SLAMMOT—The Cost of Integrating Moving Object Tracking into an EKF-SLAM Algorithm

**DOI:** 10.3390/s24175764

**Published:** 2024-09-04

**Authors:** Nicolas Mandel, Nils Kompe, Moritz Gerwin, Floris Ernst

**Affiliations:** Institute of Robotics and Cognitive Systems, University of Lübeck, 23562 Lübeck, Germanym.gerwin@uni-luebeck.de (M.G.); floris.ernst@uni-luebeck.de (F.E.)

**Keywords:** SLAM, SLAMMOT, object tracking, dynamic landmarks, EKF, filtering, Bayesian filtering

## Abstract

The treatment of moving objects in simultaneous localization and mapping (SLAM) is a key challenge in contemporary robotics. In this paper, we propose an extension of the EKF-SLAM algorithm that incorporates moving objects into the estimation process, which we term KISS. We have extended the robotic vision toolbox to analyze the influence of moving objects in simulations. Two linear and one nonlinear motion models are used to represent the moving objects. The observation model remains the same for all objects. The proposed model is evaluated against an implementation of the state-of-the-art formulation for moving object tracking, DATMO. We investigate increasing numbers of static landmarks and dynamic objects to demonstrate the impact on the algorithm and compare it with cases where a moving object is mistakenly integrated as a static landmark (false negative) and a static landmark as a moving object (false positive). In practice, distances to dynamic objects are important, and we propose the safety–distance–error metric to evaluate the difference between the true and estimated distances to a dynamic object. The results show that false positives have a negligible impact on map distortion and ATE with increasing static landmarks, while false negatives significantly distort maps and degrade performance metrics. Explicitly modeling dynamic objects not only performs comparably in terms of map distortion and ATE but also enables more accurate tracking of dynamic objects with a lower safety–distance–error than DATMO. We recommend that researchers model objects with uncertain motion using a simple constant position model, hence we name our contribution Keep it Static SLAMMOT. We hope this work will provide valuable data points and insights for future research into integrating moving objects into SLAM algorithms.

## 1. Introduction

Simultaneous localization and mapping (SLAM) has matured from a research topic to a real-world impact in the last decade [1]. While algorithms and systems have led to sophisticated solutions, the treatment of dynamic objects in SLAM is a key problem in contemporary robotics [1,2].

Having accurate information about the location of dynamic objects within the environment is essential for path planning and collision avoidance, which is key in mobile robotics applications, such as autonomous driving. Information about the state of dynamic objects like cars, pedestrians, and bicycles is necessary for safe operation.

The most common approach is to separate tracking and mapping into two phases, which was pioneered by Wang in his landmark thesis [3]. The work revolves around the assumption that dynamic landmarks do not yield useful information for self-localization of the robot and, therefore, self-localization and dynamic object tracking are separated into multiple steps (in this work, “dynamic landmark”, “moving object”, and “dynamic object” are used interchangeably).

However, in this paper, an approach that integrates dynamic landmarks into an EKF-SLAM formulation is shown; it is demonstrated that this integration leads to improved tracking and (in some cases) self-localization.

**Figure 1:**
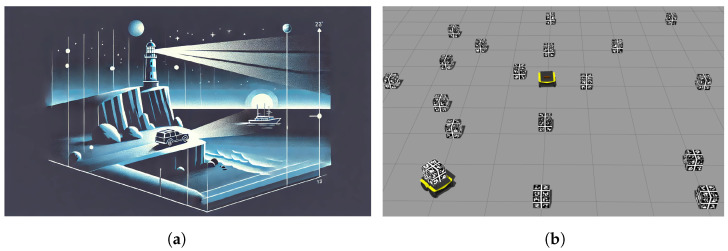
Graphical representation of the estimation problem as a simplified triangulation problem, alongside a software simulation. (**a**) The ship can estimate its own position using the lighthouse as a static landmark and the car as a dynamic landmark. (**b**) The setup in software simulations, including Gazebo, ClearPath Jackals (Kitchener, ON, Canada) as robots, and ArUco markers as landmarks. The moving agent is at the center and all marker cubes are static. A second Jackal, carrying a marker, has its path estimated by the first Jackal.

The following minimalistic thought experiment is presented in Figure 1a: A ship is positioned in front of a cliff at night, with a lighthouse on the cliff and a car on the cliff with its headlights on. In the standard SLAM scenario, the car could not be used for localization; however, in the case presented in this research, the car can contribute to the localization of the ship while its location is simultaneously being estimated alongside it. Research on SLAM predictions indicates a direct possibility of predicting the movement of dynamic objects, as shown in [4], where a deep learning module predicted moving objects to continue moving at traffic lights.

The rest of this paper is structured as follows: Initially, a background on the topic of moving object tracking (MOT) and its integration with SLAM—referred to as SLAMMOT—is provided, along with the contributions of this paper. In the next section, the mathematical and experimental setups are presented, followed by the presentation of results. Finally, our conclusions with considerations for future research are presented.

### 1.1. Background

Structure-from-motion (SfM) and visual SLAM are two closely related problems [2,5] and commonly treat dynamic objects in one of three ways: The first removes moving objects for mapping. The second only tracks moving objects and discards self-estimation. The third includes both; however, the prevailing form is to conduct SLAM first and track moving objects in a second step.

Wang [3] pioneered the third approach with the detection and tracking of moving object (DATMO) system, which separates the posteriors of moving objects from the SLAM posterior. With $x_{k}$ denoting the robot state at time *k*, *M* denoting the static map state, $O_{k}$ denoting the moving object’s state at time *k*, and $z_{k}$ denoting the observations, which are separated into object observations, $z_{k}^{o}$, and map observations, $z_{k}^{m}$, the probability distribution is as follows:

(1) $$p\left(O_{k},M,x_{k}|Z_{k},U_{k}\right)\propto p\left(z_{k}^{o}|O_{k},x_{k}\right)p\left(z_{k}^{m}|M,x_{k}\right)$$

which states that the object states only depend on the robot state at time *k*. The underlying assumption for this separation is that an integration is assumed to be computationally demanding, but that dynamic landmarks do not contribute to localization and mapping, but may also have negative effects. (We refer the interested reader to Wang [3] p. 38).

Fenwick [6] extended the EKF solution to include multiple robots collaborating in the SLAM context, analytically showing the feasibility of integrating multiple moving objects into the equations and significantly improving overall map certainty with the integration of multiple information sources.

The algorithm presented in this research builds on the algorithmic integration from Fenwick [6] and extends it to include dynamic landmarks as treated by Wang [3] by not considering information shared by dynamic landmarks, which can be seen as expanding the generalized EKF algorithm, as hinted at by Wang [3].

One of the contributors to the success of Kalman Filter algorithms in SLAM is that maintained correlations allow for updating and reducing the uncertainty of unseen landmarks. We hypothesize that this can be extended to dynamic landmarks as well [7,8]. This research presents simulations to support this assumption.

### 1.2. Related Literature

Dynamic objects require careful consideration in estimation problems [2] and contemporary research addresses this problem in multiple ways. Dynamic objects can be considered as external disturbances to the system, which are modeled in various ways in control research [9,10]. SLAM researchers have focused their efforts on selected methods for treating dynamic objects. Fenwick [6] integrates multiple moving robots into a single SLAM algorithm, in which the information shared between the robots contributes significantly to decreasing the map uncertainty. Wang [3] separated SLAM and MOT into two separate steps, reducing the problem space and laying the foundation for contemporary approaches that model dynamic objects. Sola [11] extended the DATMO approach to stereo cameras, additionally estimating the second camera’s parameters and contributing algorithmic improvements such as a transformation between time steps for improved data association. Augenstein et al. [12] modified a SLAM solution to track a dynamic tumbling object in relation to another moving object, thereby uncoupling the origin frame from a fixed world frame. Bouhlabal et al. [4] developed a depth prediction module using transformer architectures and discovered, as a side effect, that objects were predicted to move at traffic lights. Cadena et al. [1] stated that SLAM can be thought of as a mechanism to compress all past observations of the model into a task-dependent sufficient statistic and call for further research, while Skinner [13] highlighted gaps in contemporary evaluation of SLAM systems, due to the application of sampling methods.

Implementations of SLAM algorithms—alongside other fundamental robotics algorithms—are presented in the recent textbook by Corke [7]. This work not only provides code implementations for numeric simulations of many SLAM algorithms presented in the elementary textbook by Thrun et al. [14], but also updates these to use efficient linear algebraic routines. In contemporary approaches considering dynamic objects, the separation of posteriors is widely used [2], such as in the semantic approach by Rosinol et al. [15], who tracked humans in the environment in a separate posterior. In state-of-the-art computer vision, dynamic objects are modeled and predicted using deep learning models. Li and Liu [16] separated the learning step to enhance dense prediction.

While some SLAM systems have been extended to accommodate specifically deformable environments, as demonstrated by Lamarca et al. [17], our research assumes that most landmarks are static and only a limited number are dynamic, under the assumption of rigid body transformations. Qiu et al. [18] considered dynamic objects by including them as articulated objects in an offline factor graph formulation with stereo images, extending the work of Henein et al. [19]. Research on depth prediction for monocular cameras suggests the predictable behavior of dynamic landmarks [4], where dynamic objects were predicted to continue moving beyond the observed window. Henning et al. [20,21] improved camera tracking in monocular SfM by incorporating a parameterized human body model with a learned motion prediction module.

The majority of research for online algorithms hinges on the model by Wang [3], which separates self-estimation through SLAM from moving object tracking. Research in this area is active and ongoing, with motion modeled either in an object-based or grid-based approach [22]. However, to the best of our knowledge, this is the first contribution that directly integrates dynamic landmarks into an EKF-SLAM algorithm without receiving shared information from other dynamic objects.

### 1.3. Contributions

This research presents the following contributions:

1. An algorithmic integration of dynamic landmarks into the EKF-SLAM algorithm, which includes the estimation of unobserved states and is named Keep it Static SLAMMOT (KISS).
2. An implementation as an extension of the toolbox for robotic vision [23] to SLAM with multiple robots, including an implementation of DATMO [3].
3. A detailed investigation of metrics that represent the quality of the map and robot track.
4. A new safety-relevant metric for SLAMMOT, which we refer to as safety–distance–error (SDE).
5. Proposals for researchers on handling potentially dynamic landmarks.

### 1.4. Structure

The remainder of this paper is structured as follows. Section 2 presents the materials and methods used to develop this approach, including the mathematical notation and derivation, simulation settings, and metrics. Section 3 presents the results of the experiments and Section 4 places them in context and highlights special cases. Section 5 concludes with the impacts and ramifications, as well as suggestions for researchers on how to deal with dynamic objects.

## 2. Materials and Methods

### 2.1. Notation

Vectors are denoted by lowercase bold font, such as x, matrices are denoted by uppercase bold font V. Estimated values are indicated with a hat $\hat{V}$ and predicted values by a superscript + symbol: $x_{k}^{+}$. Time steps are indicated by subscripts *k* and vehicle and landmark components are indicated by a second, comma-separated subscript *v* and lm, respectively, e.g., ${\hat{x}}_{k,v}^{+}$ denotes the predicted state of the vehicle at time *k*.

### 2.2. The Generalized EKF-SLAM Algorithm

The EKF-SLAM algorithm is one of the earliest SLAM extensions to nonlinear models [14] and is commonly used for online tracking. It is governed by two phases: the prediction phase and the update phase. Wang [3] lays the foundation for the formalization of SLAM with generic objects; however, abandons further developments with the assumption that dynamic landmarks do not contribute to ego- and map estimation. Nevertheless, as explained above, this research proposes that dynamic objects can indeed contribute to localization estimation when the algorithm is generalized. This section completes that generalization.

For the sake of readability, only the EKF equations that require modification are listed here and the notation by Corke [7] is adopted and expanded to include dynamic landmarks. The complete EKF equations for modern robotics are listed in Appendix A.

Central to an EKF algorithm is the Kalman gain matrix K, which distributes the innovation of the observation to the states, as well as the covariance matrix P, which indicates the level of uncertainty. The required changes to these equations are explained in the following sections.

#### 2.2.1. Prediction Phase

During the prediction phase, the model projects the state (2) and covariance (4) of the world one time step into the future. This prediction step can be decomposed into separate predictions for the vehicle and landmarks as follows:

(2) $$x_{k+1}^{+}=f\left({\hat{x}}_{k},u,\sigma \right)=\left[\begin{array}{c} f_{v}\left({\hat{x}}_{k,v},u_{k},\sigma \right)\\ f_{lm}\left({\hat{x}}_{k,lm}\right) \end{array}\right]$$

with ${\hat{x}}_{k}$ representing the current state estimate, which includes the vehicle states ${\hat{x}}_{k,v}$ and map state estimates ${\hat{x}}_{k,lm}$; u representing the odometry vector, and σ denoting the process noise, which is applied to the odometry and is combined to form matrix Σ. The vehicle prediction equations are detailed in Appendix A.1. Static landmarks are projected with an identity function:

(3) $$x_{k+1,lm}^{+}=f_{lm}\left({\hat{x}}_{k,lm}\right)=\left[\begin{array}{c} {\hat{x}}_{k,s}\\ {\hat{y}}_{k,s} \end{array}\right]$$

The covariance is predicted with the following:

(4) $${\hat{P}}_{k+1}^{+}=F_{x}{\hat{P}}_{k}{F_{x}}^{T}+F_{\sigma }\hat{\Sigma }F_{\sigma }^{T}$$

where $F_{x}$ and $F_{\sigma }$ are the Jacobians w.r.t. the state x and the process noise σ, assumed to be linearly independent. The landmarks and vehicle are assumed to be independent [3]; therefore, Jacobians are composed block-wise:

(5) $$F_{x}=\left[\begin{array}{ccc} F_{x_{v}} & \ldots & 0\\ \vdots & \ddots & \\ 0 & \; & F_{x_{lm}} \end{array}\right]$$

and

(6) $$F_{\sigma }=\left[\begin{array}{ccc} F_{\sigma_{v}} & \ldots & 0\\ \vdots & \ddots & \\ 0 & \; & F_{\sigma_{lm}} \end{array}\right]$$

The vehicle Jacobians, $F_{x_{v}}$ and $F_{\sigma_{v}}$, are listed in Appendix A.1.

Equation (3) yields Jacobians for the static landmark states, which are assumed to be absolutely stationary, as follows:

(7) $$F_{x_{s}}=I_{2\times 2}$$

and

(8) $$F_{\sigma_{s}}=0_{2\times 2}$$

which, under inspection of (4), show that the predicted uncertainty is equal to the updated uncertainty, as the identity matrix multiplication results in ${\hat{P}}_{k+1}^{+}={\hat{P}}_{k}$ for static landmarks. $F_{\sigma_{s}}=0_{2\times 2}$ ensures that no uncertainty is added through the process model in the second term of (4), regardless of model noise $\hat{\Sigma }$.

Equations (7) and (8) are modified for dynamic landmarks. The prediction Equation (3) is not an identity function anymore, but depends on the motion model presented in Appendix B, which also changes the Jacobians.

Landmarks are assumed to move independently of each other, preserving the block-diagonality of (5) and (6).

#### 2.2.2. Update Phase

During the update phase, measurements of the outside world are incorporated to correct the predictions. A range and bearing sensor resembling a LIDAR scanner is modeled in this work and detailed in Appendix A.2.

The Kalman gain tailors the degree to which updates are distributed to the state *x* and covariance *P*, as follows:

(9) $$K=P_{k+1}^{+}H_{x}^{T}{\left(H_{x}P_{k+1}^{+}H_{x}^{T}+H_{\omega }\hat{\Omega }H_{\omega }^{T}\right)}^{-1}$$

with the sensor noise model detailed in Appendix A.2. The Kalman gain distributes the innovation ν from (A12) to the state updates, shown in (10), and tailors the degree to which uncertainty is subtracted from the covariance matrix; see (11).

(10) $${\hat{x}}_{k+1}={\hat{x}}_{k+1}^{+}+K\nu$$

(11) $${\hat{P}}_{k+1}=P_{k+1}^{+}-KH_{x}P_{k+1}^{+}$$

The Kalman gain is determined by combining the predicted uncertainty from (4) and the estimated sensor noise model $\hat{\Omega }$ (A13) with Jacobians of (A11), $H_{x}$, and $H_{\omega }$, which differentiate w.r.t the state x and the noise ω. $H_{w}$ is an identity matrix, $H_{w}=I_{2\times 2}$, due to additive noise in (A11). Equation (A11) depends on the robot state $x_{v}$ as well as the landmark state $x_{lm}$, and is constructed by differentiating w.r.t both. For a single landmark, the update Jacobian is as follows:

(12) $$H_{x,z}=\left[\begin{array}{c} H_{v,z}\ldots 0\ldots H_{lm,z}\ldots 0\ldots \end{array}\right]$$

with the subscript *z* denoting the corresponding observation. The full observation Jacobian $H_{x}$ is a row-stack of these, analogous to (A24), with the standard Jacobians $H_{v}$ and $H_{lm}$ listed in Appendix A.2.

### 2.3. Extension to Dynamic Landmarks

Dynamic landmark states are incorporated by altering the prediction function $f_{lm}\left({\hat{x}}_{k,lm}\right)$, part of (2), and including its Jacobians $F_{\sigma_{lm}}$ and $F_{x_{lm}}$ into (6) and (5) as submatrices, respectively.

Projection equations for state (2), vehicle (A2), and covariance (4), as well as the construction of summary Jacobians (5) and (6) remain untouched.

Three different motion models are included as part of this work. The overwritten prediction equation $f({\hat{x}}_{k+1,lm})$ (3) and Jacobians $F_{x_{lm}}$ (7) and $F_{\sigma_{lm}}$ (8) are described in the following appendices:

1. A static linear motion model, which models noise as the only source of motion and assumes independence between the two motion plane dimensions, Appendix B.1
2. A linear kinematic motion model, which models linearly independent velocity as the source of motion and assumes independence between the two motion plane dimensions; Appendix B.2
3. A nonlinear kinematic motion model, specifically a bicycle model, is used to model the changes between the two motion plane dimensions through changes in velocity and angle as detailed in Appendix B.3

It should be noted that both kinematic motion models include states that are not observed but are nonetheless estimated by the filter through changes in the observable x and y changes.

Observation functions require minimal alterations to keep consistency with state length indices, as explained in Appendix C. Note that both kinematic models include states that cannot be observed by the sensor, however, can still be estimated by the algorithm. This is achieved by altering $H_{lm}$ (A14), such that it extends to unseen states.

The proposed approach is named “KISS”—Keep it Static SLAMMOT—for the remainder of this work.

### 2.4. Experiments

Simulations allow for reducing the impact of potential erroneous real-world influences. This paper focuses on numerical simulations to expose the effects of the model’s choice on the algorithm performance while reducing conflating biases introduced through experimental influences such as data association, re-identification, loop closure, and time synchronization [1].

Simulations also allow for unequivocal identification of static and dynamic landmarks, thereby allowing for a clear distinction between true and false models and assessing the impact of false-negative and false-positive model association.

The software expands on the robotics toolbox by Corke and Haviland [23] and is made publicly available. It follows common conventions for good research code [24] and facilitates the configuration of multiple experiments through the Hydra package [25]. Experiments can be conducted with various seeds and configurable settings, such as the number of static and dynamic landmarks, or sensor distance and angle. Logs from the simulation are stored and can be reloaded using integrated scripts for full reproducibility. Additionally, a summary spreadsheet generated during simulations can be loaded into scripts to produce summary statistics.

During simulations, all filters are run with the exact same inputs, thereby allowing direct comparison of performance. Since filter calculations are deterministic and sampling is only used during simulation, all experiments are fully reproducible with these logs.

A few simplifying assumptions are made for the sake of showing the impact of the dynamic landmarks on the algorithm itself. The assumption that data association [26] is solved is made to reduce the conflating impact of data association, which is a significant problem in real-world experiments. An a priori setting provided to each filter dictates which landmark identities are treated as dynamic or static. All motion models are based on the bicycle model by Corke and Haviland [23] and paths are random according to the seed within a defined workspace. All landmarks are visible to the sensor for the entire duration of the run. Moreover, 60-s runs are used. We encourage the interested reader to access the provided code and software and provide feedback.

### 2.5. Evaluation

A standard SLAM filter does not consider dynamic landmarks, which is the fundamental baseline implemented in this research and is denoted as an “exclusive” filter. As a counterexample, a filter is implemented that considers the dynamic landmarks as static in its estimation; it is denoted as an “inclusive” filter.

#### 2.5.1. DATMO

Most contemporary approaches to modeling dynamic landmarks in SLAM are based on the work of Wang [3,22], which assumes that dynamic landmarks do not contribute to the localization of the robot, hence, combining the exclusive filter with the independent estimation of each dynamic landmark.

Sola extends the work for stereo-camera estimation [11] and introduces an additional function j, which maps objects from the robot frame of *k* to k+1 and depends on the last estimated object state ${\hat{O}}_{k}$ and the control input $u_{k}$ of the robot ([11] pp. 152, 154), as follows:

(13) $$O_{k+1}^{+}=j\left({\hat{O}}_{k},u_{k}\right)$$

and its Jacobians are used to transfer the covariance spaces. This work is reimplemented (to the best of our capabilities) without the model selection part of DATMO [3], but integrating j (13) and its Jacobians to allow for fair comparison.

Other algorithms, such as unscented Kalman filters, particle filters, and graph-based solutions [14], have achieved higher accuracies in SLAM-based solutions. To the best of our knowledge, the method of separating the posterior s for SLAM and MOT, as proposed by Wang [3], is the only comparable EKF-based approach. Modifying algorithms could confound the impacts of modeling and algorithmic changes; however, this should be considered in future research.

#### 2.5.2. Experiments

Regarding the impact of dynamic landmarks on the algorithms themselves, three primary questions are investigated:

1. What happens when a dynamic landmark is estimated as static, further referred to as a false negative?
2. What happens when a static landmark is estimated as dynamic, further referred to as a false positive?
3. What happens to overall metrics when dynamic landmarks are included inside the algorithm?

As secondary research objectives, the influences of occlusions and changing velocities are investigated independently. Occlusions are modeled by reducing the sensor range, thereby filtering any observations beyond a specified sensing range. Changing velocities are modeled by adapting the velocity depending on the heading angle, slowing dynamic objects down with larger turn angles. Certain metrics (as follows) are used to assess the impact of dynamic landmarks.

#### 2.5.3. Metrics

The quality of the trajectory of the robot is commonly evaluated on the absolute trajectory error (ATE) [13]:

(14) ATE(X,X^)=1n∑k=0n−1∥xk,v−R(x^k,v)+c∥212

where $x_{v}$ and ${\hat{x}}_{v}$ are the states corresponding to the true and estimated robot positions, respectively, and are column-stacked together over time indices *k* to form matrices X and $\hat{X}$. R and c are the rotation and translation between the estimated and true maps. They are determined through a linear least squares estimate for known correspondence [27]. Only landmarks presumed static by the model are included in the calculation. For example, a filter that incorrectly assumes landmark ten as dynamic will exclude it from the estimation. The magnitudes of the parameters serve as indicators of the spatial distortion within the estimation model.

While the aforementioned metrics evaluate summary metrics for all algorithms, a safety-relevant factor for dynamic object tracking during execution involves the distance between the robot and the dynamic landmark.

This project advocates for the use of the safety–distance–error (SDE), which calculates the difference between the true and estimated distance between the robot and the landmark. The metric is applied to the trajectory without using the transform R and c estimated through linear least squares [27], as the transform is not available during runtime. Wrong associations can lead to large map distortions, as shown by Neira [26] and illustrated in Section 3.1; therefore, the distance difference without correction is key. The SDE is calculated as the mean of the sum of squares difference between the estimated and true distances:

(15) SDE(Xv,X^v,Xlm,X^lm=1n∑k=0n−1(∥x^k,lm−x^k,v)∥−∥xk,lm−xk,v∥)212

where matrices X are the corresponding vectors column-stacked together over time indices *k* and lower values indicate better performance.

## 3. Results

This section shows the results of the simulations. Plots showing the performance across different metrics of different filters introduced in Section 2.5 contain mean and 95% confidence intervals of 20 runs with different random seeds. Selected singular cases are presented to highlight distinct algorithm properties. The following five filters are used:

1. The exclusive filter—abbreviated as EXC in the figures and colored green—excludes dynamic landmarks.
2. The inclusive filter—abbreviated as INC in the figures and colored orange—models dynamic landmarks as static.
3. The false positive filter—abbreviated as FP in the figures and colored blue—models static landmarks as dynamic.
4. The DATMO implementation presented in Section 2.5.1—abbreviated as DATMO in the figures and colored yellow—separates SLAM and MOT.
5. The KISS implementation—abbreviated as KISS in the figures and colored magenta—includes dynamic landmarks in the state estimation.

As DATMO separates SLAM and MOT, the map and ego-track of the robot are identical to the exclusive filter, and dynamic landmark estimates are added as additional metrics.

**Figure 2:**
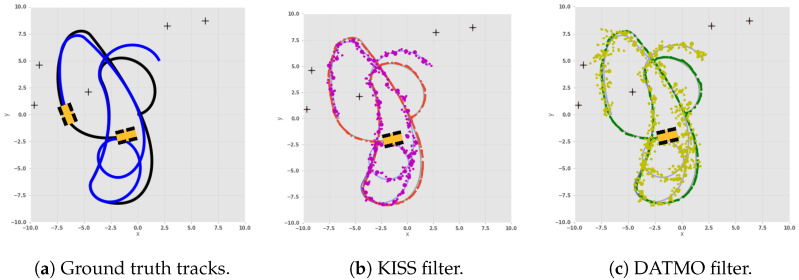
One dynamic and five static landmarks from a single run assuming a static model, as detailed in Appendix B.1; (**a**) shows the true tracks of the robot in black, the dynamic landmark in blue, and the locations of landmarks; (**b**) the estimates of the KISS approach detailed in this work; and (**c**) the estimates of the approach using the DATMO baseline. Both approaches have identical inputs.

Figure 2 shows the true tracks, as well as the estimated tracks of the robot itself and the dynamic landmark. Figure 2a shows the ground truth trajectory of both robots, with their final position denoted by the robot marker. Figure 2b shows the estimate from our model and Figure 2c from DATMO. It can be observed that the dynamic landmark estimates by DATMO vary around the true track by a much larger magnitude, while our model deviates less.

Further details on the variances in estimates are provided in Section 4.

### 3.1. False Negatives

**Figure 3:**
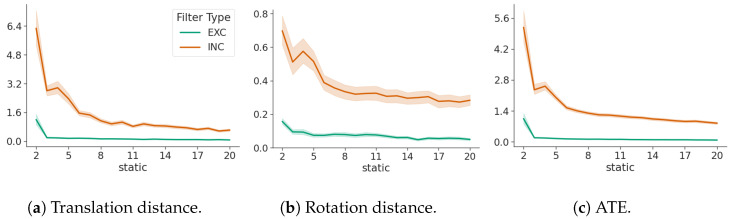
The influence of a single false negative on the translation, rotation, and ATE, respectively. Green denotes the exclusive filter “EXC” and orange the inclusive filter “INC”. The x-axis shows an increasing number of static landmarks.

Figure 3 shows that a false negative (dynamic landmark falsely assumed to be static) highly distorts estimates. The ATE is higher than the baseline, as well as map distortion parameters R and c (14). The rotation angle for the presented 2D case is extracted from the rotation matrix R and displayed. This indicates that distortion is induced by landmarks that are dynamic and falsely assumed to be static and that an increasing number of static landmarks can serve to reduce the impact, but the effect remains even with a large number of static landmarks.

### 3.2. False Positives

**Figure 4:**
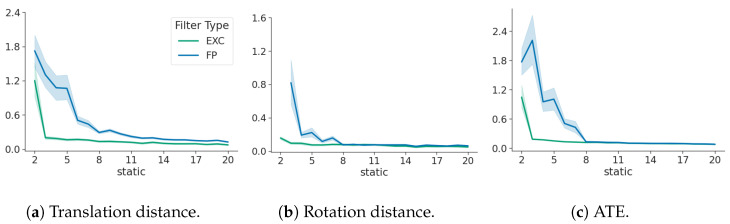
The influence of a false positive on the translation, rotation, and ATE respectively. Green denotes the best-case exclusive filter and blue denotes the false positive filter. The x-axis shows an increasing number of static landmarks.

Figure 4 shows that false positives (static landmarks falsely assumed to be dynamic) have a limited effect on the tracking performance and map distortion with an increasing number of static landmarks. The initial offset in ATE diminishes when more static landmarks are correctly visible, while translation and rotation are constantly within the same range. No distinct trend can be observed with changing motion models; therefore, they are omitted for brevity.

**Figure 5:**
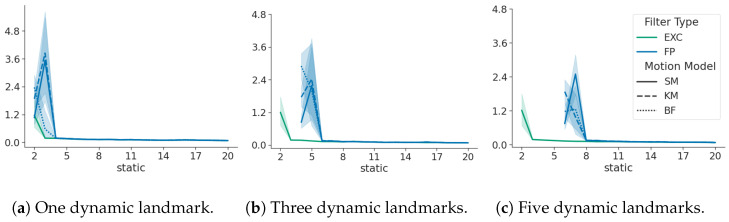
The impact of multiple false positives on ATE. For each plot, the number of static landmarks used in the simulation remains constant, but more landmarks are modeled as dynamic inside the false positive filter, causing the shift to the right. Green denotes the best-case exclusive filter and blue denotes the false positive filter. The line styles show different motion models, as explained in Appendix B. The x-axis shows an increasing number of static landmarks.

Figure 5 shows ATE with an increasing number of static landmarks, where some static landmarks are assumed to be dynamic, i.e., a false positive. With each plot, more of the fixed numbers of static landmarks are assumed to be dynamic by the false positive filter. When a static landmark is treated as dynamic, it is also excluded from the calculation of the map transform; therefore, ATE only commences when the number of static landmarks considered as static is two or greater (two is the minimum number to calculate a transform in 2D), which causes the estimates to shift to the right with increasing dynamic landmarks.

It can be observed that ATE converges after an initial large divergence, which means that with an increasing number of true static landmarks, the performance approaches the exclusive filter. This means that the impact of false positives has negligible influence on the filter performance with an increasing number of static landmarks. When compared with the false negative filter shown in Figure 3, it becomes evident that map scales are preserved when considering static landmarks as dynamic and that the impact on tracking performance is marginal.

### 3.3. Tracking Dynamic Objects

**Figure 6:**
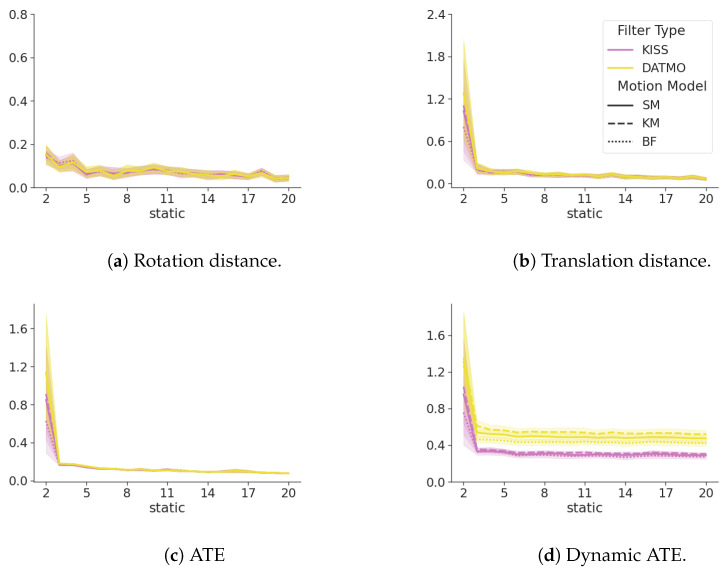
A comparison between KISS in magenta and DATMO in yellow for a single dynamic landmark. DATMO and KISS are closely related in terms of the ATE, translation distance, and rotation distance. The ATE of the dynamic landmark shows lower (better) values for KISS. The line styles show different motion models, as explained in Appendix B. The x-axis shows an increasing number of static landmarks.

Figure 6 shows a summary of the metrics between the exclusive filter, DATMO as its extension (Section 2.5.1), and KISS. All metrics are closely related and within each other’s standard deviation bounds. However, the dynamic ATE shows large differences. DATMO consistently shows a larger error in tracking the dynamic landmark, with the exact same motion models, while even the most naive model in KISS, a constant position model (Appendix B.1), surpasses DATMO with a much lower confidence interval. At the same time, differences in ATE as seen in Figure 6c, are negligible.

**Figure 7:**
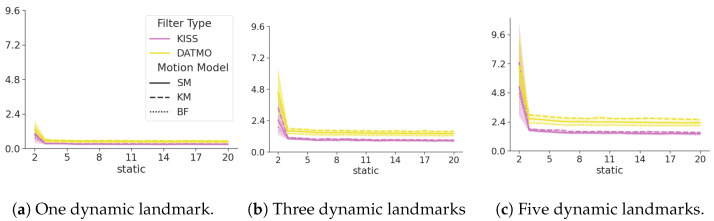
The cumulative ATE over an increasing number of static landmarks on the x-axis. Each figure increases the number of dynamic landmarks in the environment. Yellow lines denote the baseline DATMO filter, and magenta denotes the KISS filter. Smaller numbers are better.

Figure 7 shows that the cumulative ATE for dynamic objects of KISS is consistently lower than for DATMO. This difference is becoming more pronounced for an increasing number of dynamic landmarks and is valid for all motion models. Even a naive constant position model, see Appendix B.1, surpasses the performance of DATMO by a large margin, with less overlap between confidence interval bounds as the number of dynamic landmarks increases.

Table 1 shows the mean and standard deviations of the dynamic landmark ATE (14), divided by the number of dynamic landmarks. For every motion model, KISS outperforms DATMO (even simple models perform better than DATMO). The most accurate model for DATMO, the body frame model (see Appendix B.3), which is a direct implementation of the true bicycle model, is worse than the simplest static model (see Appendix B.1). The results are consistent when increasing the number of dynamic landmarks.

The kinematic model appears to perform worse than the static model, while the body frame model performs best. The body frame model is closest to the true motion of the landmark, while the static model does not estimate hidden states and does not have to distribute information to hidden states, a possible explanation for the slight difference in performance.

### 3.4. SDE

**Figure 8:**
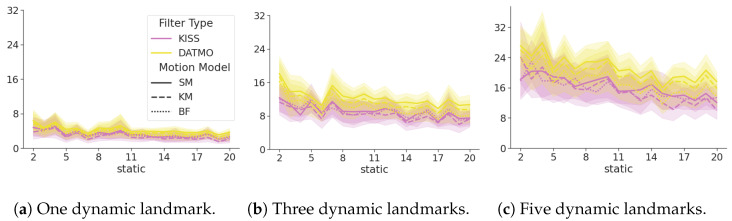
SDE (15) of KISS in magenta, DATMO in yellow. Different motion models for the dynamic landmarks are denoted by line styles. Lower values are better. The error of KISS is consistently lower than the error of DATMO. With an increasing number of dynamic landmarks, this trend becomes more pronounced. The error is consistent with an increasing number of static landmarks.

Figure 8 shows the SDE (15) of the DATMO and the KISS filter in yellow and magenta, respectively. The line styles show the different motion models for the dynamic objects. The number of dynamic landmarks increases with each subplot, and the x-axis denotes the number of static landmarks in the environment. Lower values indicate smaller errors and, hence, better performance. The SDE of KISS is consistently lower than that of DATMO. This difference becomes more pronounced in absolute terms with an increasing number of dynamic landmarks, while remaining constant with an increasing number of static landmarks.

### 3.5. Occlusions

Occlusions are modeled by reducing the sensor range and filtering out values that are further away. With map dimensions of 20 × 20, this greatly reduces the amount of observed landmarks at each time step. For brevity, only the results for 5 are presented here, while the results for 10 can be found in Appendix D and the publicly available data.

**Figure 9:**
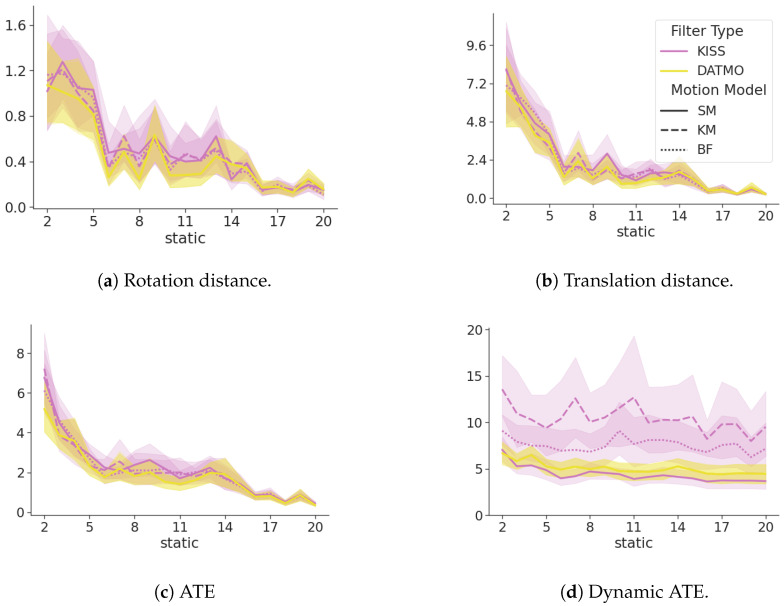
A comparison of KISS in magenta, DATMO in yellow for a single dynamic landmark, and the sensing range reduced to 5. DATMO and KISS are closely related in terms of ATE, translation, and rotation distance. The ATE of the dynamic landmark shows lower (better) values for KISS with a static model, while errors increase for kinematic models. The line styles show different motion models, as explained in Appendix B. The x-axis shows an increasing number of static landmarks.

Figure 9 shows the impact of reducing the sensing range, which excludes some tracked landmarks from each observation. While ATE, rotation, and translation distance exhibit similar behavior to full observability, as seen in Figure 6, dynamic ATE is greatly reduced for kinematic models.

This effect can most likely be attributed to a reduced number of observations per time step, with an increased number of states to track, greatly reducing the amount of information available at each time step to the filter, which additionally attempts to estimate multiple hidden states.

### 3.6. Changing Velocity

**Figure 10:**
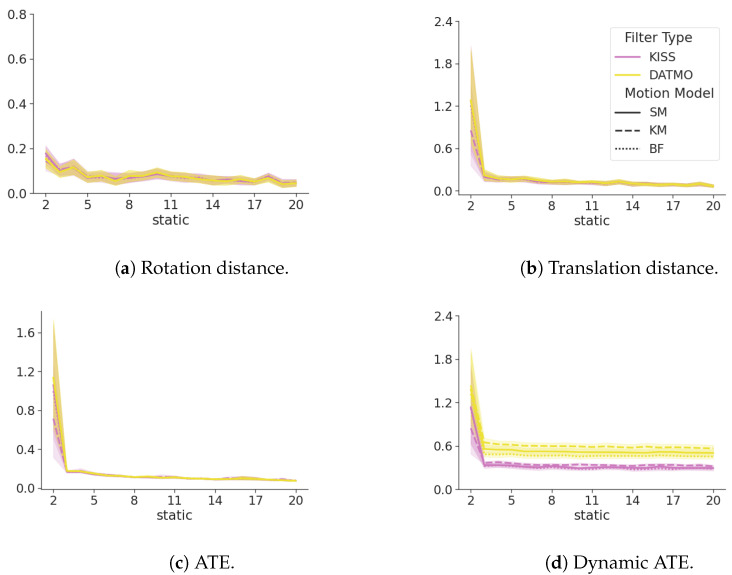
A comparison between KISS in magenta and DATMO in yellow for a single dynamic landmark and changing velocities, depending on the heading angle. DATMO and KISS are closely related in terms of ATE, translation, and rotation distance. The ATE of the dynamic landmark shows lower (better) values for KISS with a static model, while errors increase for kinematic models. The line styles show different motion models, as explained in Appendix B. The x-axis shows an increasing number of static landmarks.

Figure 10 shows the results of KISS and DATMO when modifying the velocity of the moving object depending on the heading angle. Velocities vary between 0.24 and 1 during simulations, while the velocity of the primary object remains the same. When compared with Figure 6, it can clearly be observed that a changing velocity does not impact the filters in different manners.

### 3.7. Uncertainty

**Figure 11:**
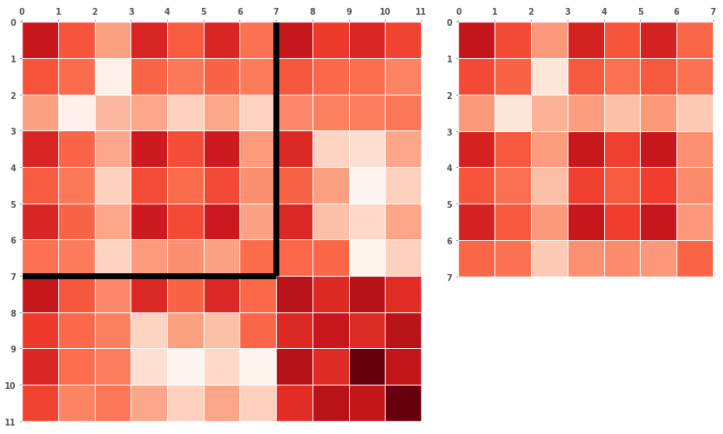
The logarithm of the covariance matrices at the last time step for the simulation of two static markers and one dynamic with seed zero. Darker values indicate a higher correlation. The right side shows the exclusive filter, while the left shows KISS with a linear kinematic motion model; see Appendix B.2. The exclusive is a subset in the kinematic model, with four rows and columns appended with the values of the kinematic landmark.

Figure 11 shows the base ten logarithms of the covariance matrices at the last time step for the filter, including the dynamic landmark in a kinematic model on the left, as well as the filter excluding dynamic landmarks on the right. The matrix on the right is fully contained in the matrix on the left. The left has four added rows and columns, which correspond to the four states of the dynamic landmark. The four values exhibit a large autocorrelation, as well as a large correlation with the first three states, which correspond to the vehicle. All other values are lower, but not zero.

This shows that the dynamic landmark is indirectly correlated to static landmarks through its relationship with the vehicle, to which it is highly correlated. This is a common property of EKF-SLAM, where observations of one landmark can be used to correct an associated landmark, as is happening within the algorithm here.

It is also clearly visible that the two unobserved states can be estimated. This capability is a general benefit of the EKF algorithm, which allows for estimating unobserved states through correlations introduced by the models. The motion model outlined in Appendix B.2 only allows motion through velocities; therefore, any change in position must be caused by velocities, as indicated by the large autocorrelations of the landmark states. The non-zero states in the additional columns and rows, introduced over time, show a correlation between the dynamic and static landmarks.

When all landmarks are assumed to be dynamic, there is no certainty of a fixed frame shared between the robot and all dynamic landmarks, reducing the calculations to a form of extended dead reckoning [7], with ever-increasing uncertainty since no steadfast anchor with 0-uncertainty can be used to fix poses. However, in some cases, this may be desirable as shown by Augenstein [12], who assumed the fixed frame to be the robot frame, which in itself is allowed to move.

## 4. Discussion

Figure 3 clearly shows that falsely considering a dynamic landmark as static without any leeway for motion through noise has a significant impact on all metrics, which cannot be recovered even by correctly including more static landmarks. Figure 5 shows that with an increasing number of static landmarks, the effect of falsely estimating a static landmark as dynamic on ATE is negligible.

Figure 4 also shows that R and c converge to the values of the exclusive filter with an increasing number of static landmarks. This indicates that initializing a landmark as dynamic can be seen as a conservative measure to maintain map deformation and metric consistency.

The differences in estimating the dynamic landmarks between KISS and DATMO are substantial, as seen in Figure 2. To investigate this impact, a specific case was inspected, where the difference between ${\hat{x}}_{k,lm}$ and ${\hat{x}}_{k+1,lm}$ was the largest for a dynamic landmark, *lm*.

Figure 2 shows that DATMO estimates highly vary around the true path, while KISS estimates are much closer to the true path. Further investigations of specific cases with the largest discrepancy between *k* and k+1 yield potential insights into the behavior.

**Figure 12:**
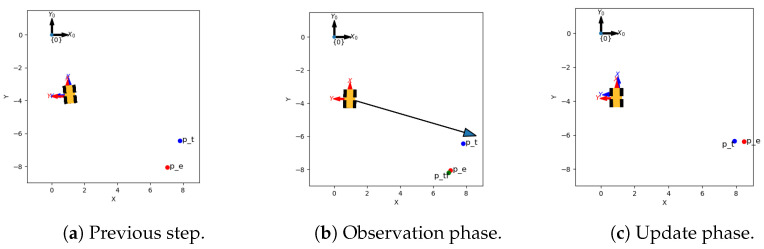
The figure shows the steps between *k* and k+1 for a single dynamic landmark denoted as a point and the robot by the symbol. True poses are represented in blue and estimated in red. The green dot represents the frame transform explained in Section 2.5.1. The estimate in (**a**) is off by a large margin. The observation generated by the robot in (**b**) causes a large shift toward the true position, visible in (**c**).

Figure 12 shows DATMO’s true and estimated robot pose as coordinate systems, with the true and estimated moving landmark positions as dots. The observation is denoted by an arrow. The blue coordinate system denotes the true pose, and the red coordinate system the estimates. The estimate, which deviates from the true pose in Figure 12a, causes the predicted value of the dynamic landmark to move even further from the true pose. This is due to the transformation of the point into the new frame, as detailed in Equation (13), shown by the green dot in Figure 12b ([11] pp. 152, 154). The arrow, denoting the observation for the said landmark, is noisy and past the true point position and yields a large update for the landmark in Figure 12c, which is very close to the true value in the next step.

Cases like this explain the large—and unrealistic—changes in estimates of dynamic landmarks. The pose of the dynamic landmark is estimated assuming the pose of the robot is true. Hence, if the position of the robot is off in one direction and the estimate of the dynamic landmark is also off in the same direction, this error accumulates into a second-degree error. Observations about the dynamic landmark cannot be used to update the robot pose and vice-versa.

The additive nature of the separation of the posteriors for estimating the true position causes large jumps in the estimates by DATMO, while KISS is capable of combining both estimates to correct both poses, incorporating the additional information and potentially yielding self-accuracy for the sake of more accurate dynamic landmark localization.

While this appears to be a downside, as the trajectory may suffer, the upside is that additional information can be used in the estimation process. Furthermore, the major concern for dynamic landmarks is the distance between the robot and dynamic landmarks, which is summarized in the SDE (15). Figure 8 shows that the error in the safety distance of KISS is consistently lower than DATMO, showing that the estimate of the distance to dynamic objects is superior to estimating dynamic objects independently.

The real-time capability and computational complexity of EKFs are dictated by the update of the covariance matrix (9), either from the matrix inversion, which can—at best—be completed in O(k^2.8^) time [3,14], where k is the dimension of the measurement vector z (A11) or the various matrix multiplications, which are quadratic in the number of states, n: O(n^2^). Conventionally, the state space is much larger and dominates the complexity [14]. When integrating dynamic objects, n and k are not only composed of the robot state and static landmarks but also of dynamic landmarks. The constant position motion model, Appendix B.1, adds two states per dynamic landmark, which simplifies index handling and minimizes the increase in computational complexity. However, tracking objects that are far beyond the sensing range and may not reappear, as opposed to static objects, only adds computational complexity.

The increased computational complexity for kinematic models, as well as reduced accuracy, as shown in Figure 9d, shows that unobserved dynamic objects may potentially have a negative impact on state estimation and tracking and that further research is necessary to establish under which circumstances integration should avoided.

Further simulations and software-in-the-loop simulations should elucidate this connection and validate it in real-world experiments.

## 5. Conclusions

This research has shown that it is possible to integrate dynamic landmarks into the EKF-SLAM algorithm, which is called KISS throughout this work. While common and safety-relevant metrics consistently improve when directly integrating dynamic landmarks, there is always a cost. The cost, in this case, is a high degree of correlation between the robot and the dynamic landmarks, as shown in Figure 11, with slight increases in computational complexity and negative impacts in reduced sensing ranges; see Figure 9d. Increased accuracy in estimating the state of the dynamic landmark is only possible through these correlations, and potentially negatively impacts the trajectory estimation.

However, with an increasing number of static landmarks, the effect of including more static landmarks into the self-estimation shows diminishing returns and the information can effectively be propagated through to the dynamic landmark to improve its estimate.

The integration of dynamic landmarks can be regarded as somewhere in between the standard SLAM case with only rigid landmarks [7,14], DATMO and the collaborative SLAM case, as presented by Fenwick [6]. However, in contrast to Fenwick, there are no observations from the collaborator to the mapping instance, which would reduce uncertainty beyond the single-actor SLAM case. This also enables its use outside of classic SLAM applications, such as virtual reality (VR), to enable a more accurate and stable mapping of the environment in dynamic settings, possibly enhancing the user experience.

### Recommendations for SLAM Researchers

Figure 3 and Figure 4 show that it is clearly beneficial to falsely consider a landmark as dynamic rather than static. Map distortion and ATE are unrecoverable through more static landmarks, once falsely assumed as static, and vice-versa—more static landmarks can reduce the effect of falsely assuming a static landmark as dynamic.

More accurate motion models improve the estimates, but not by a large margin as shown in Figure 7 and Table 1; therefore, we suggest that researchers use the simplest approach in case of uncertainty, a static model presented in Appendix B.1, which not only appears to work reasonably well, but also does not require extensive index management in the EKF matrices.

While using more accurate, kinematic, motion models, such as a linear kinematic version, Appendix B.2, or a nonlinear-kinematic version, Appendix B.3, may improve the estimates, in practice, the validity of the model is difficult to obtain.

Modeling noise as the only source of movement, as shown in Appendix B.1, is a naive and conservative approach, however, it is effective at mitigating errors introduced by unwanted movement in the absence of better knowledge and does not introduce additional hidden states requiring estimation; therefore, we propose resorting to this model in the case of uncertainty.

In practice, it is possible to model the majority of dynamic actors in a conventional roadside setting with the three models presented in Appendix B. Vehicles, such as cars, trucks, or cyclists, are most accurately modeled as nonlinear kinematic actors, as seen in Appendix B.3, while pedestrians, who can sidestep and generally have much lower velocities, should be modeled with a linearly independent model, as detailed in Appendix B.1 or Appendix B.2. Parking cars, while they could move, are unlikely to do so, and their state can be modeled with a constant position model (see Appendix B.1), just like other potentially movable objects within small spaces, such as roadside branches or leaves. However, since data association is challenging, a simple and conservative approach would be to model all uncertain objects with a constant position, allowing motion through the noise, as outlined in Appendix B.1, which does not introduce hidden states, which cannot be observed, to be estimated.

This proposal is the reason this research has been named KISS.

If dynamic landmark estimates are not necessary, it is always best to reliably exclude dynamic landmarks. This approach ensures that the full available information contributes to the own pose estimate; otherwise, the information will be distributed among all dynamic components of the estimation problem.

Additionally, the greater the number of reliably static landmarks, the more accurate the overall estimate will be, regardless of motion models or false positives.

While the overall accuracy of the EKF-SLAM algorithm has long been surpassed by other solutions, such as pose graph formulations, its online capability allows estimation results to be available iteratively at time step k. Further developments could extend to other formulations such as graph-slam solutions or particle-filter solutions.

This research serves as an analysis of the impact on algorithmic performance, but it is not exhaustive. Further work on the impact of reduced sensor models, different noise-generating models [28], and different vehicle and odometry models is necessary. Conventional research topics in SLAM, such as data association [26], pose graph solutions [14], loop-closure or semantic SLAM [1], as well as issues arising from real-world applications, also apply to the proposed algorithm and require further investigation. Research on SLAM predictions indicates that there is a direct possibility of predicting movement, as shown in [4], where moving objects are predicted at lights by a deep learning module.

Factor graph solutions can incorporate dynamic rigid-body motion [18,19,20,21]; however, this research is the first step toward demonstrating that online SLAM algorithms can also benefit from the direct integration of dynamic landmarks. Further research should aim to elucidate the connection between the integration of dynamic landmarks and accuracy metrics and validate the applicability in real-world experiments. We hope this research will inspire further efforts to integrate dynamic landmarks directly into SLAM. By making the software package and all result files publicly available, we aim to encourage feedback, facilitate further algorithmic comparisons, and promote additional developments.

## Figures and Tables

**Table 1:** Mean and standard deviations of normalized dynamic ATE for 15 static landmarks. The cumulative ATE is divided by the number of dynamic landmarks for comparison.

Dynamic Landmarks		1		2		3		4		5	
Dynamic Model	Filter	Mean	std	Mean	std	Mean	std	Mean	std	Mean	std
BF	DATMO	0.42802	0.07463	0.4244	0.07054	0.42383	0.06123	0.42031	0.0523	0.4208	0.04262
	KISS	**0.27766**	0.06172	**0.2763**	0.05591	**0.2863**	0.05816	**0.2792**	0.04328	**0.27931**	0.03521
KM	DATMO	0.52586	0.1088	0.53724	0.109	0.52225	0.08444	0.52417	0.06736	0.53813	0.06897
	KISS	**0.30936**	0.04347	**0.31145**	0.03754	**0.312**	0.03804	**0.31913**	0.04925	**0.3164**	0.03525
SM	DATMO	0.48162	0.09204	0.47692	0.09231	0.4746	0.07746	0.46957	0.06724	0.46934	0.05298
	KISS	**0.29757**	0.04213	**0.2938**	0.04099	**0.29051**	0.03116	**0.2951**	0.03733	**0.28976**	0.02416

**Figure A1:**
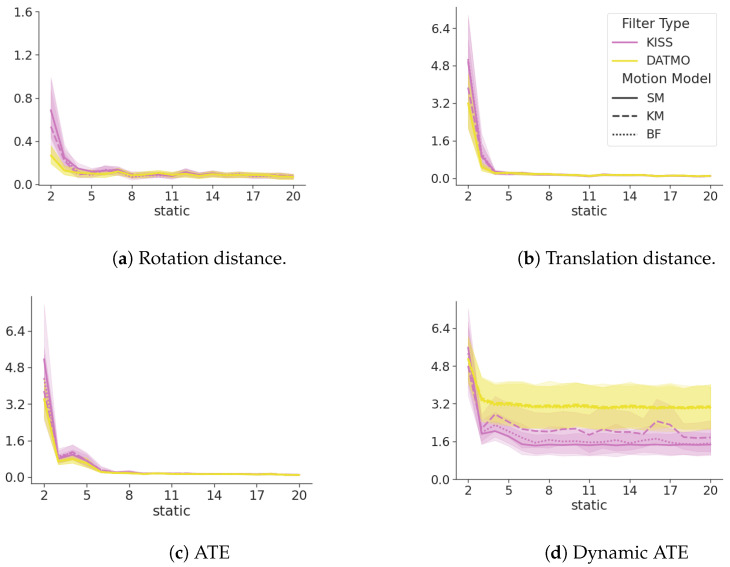
A comparison of KISS in magenta and DATMO in yellow for a single dynamic landmark with a sensing range reduced to 10. DATMO and KISS are closely related in terms of ATE, translation, and rotation distance. The ATE for the dynamic landmark exhibits lower (better) values for KISS using a static model, while errors increase for kinematic models. The line styles represent different motion models, as explained in Appendix B. The x-axis shows an increasing number of static landmarks.

## Data Availability

Code available at https://github.com/NicoMandel/mrekf_slam. Data available at https://srv01.rob.uni-luebeck.de/~mandel/downloads/ (accessed on 1 September 2024).

## Abbreviations

The following abbreviations are used in this manuscript:

EKF	extended Kalman filter
SLAM	simultaneous localization and mapping
SfM	structure-from-motion
MOT	moving object tracking
KISS	Keep it Static SLAMMOT
DATMO	detection and tracking of moving objects
ATE	absolute trajectory error
SDE	safety distance error

## Appendix A. EKF Equations

The prediction step can be decomposed into a prediction for vehicles and landmarks:

(A1) $$x_{k+1}^{+}=f\left({\hat{x}}_{k},u,\sigma \right)=\left[\begin{array}{c} f_{v}\left({\hat{x}}_{k,v},u_{k},\sigma \right)\\ f_{lm}\left({\hat{x}}_{k,lm}\right) \end{array}\right]$$

with ${\hat{x}}_{k}$ denoting the current state estimate, $u_{k}$ denoting the control input, and σ denoting the process applied to the odometry, these are combined to form the matrix Σ.

### Appendix A.1. Vehicle Model and Jacobians

The vehicle is predicted with the following:

(A2) $$f_{v}\left({\hat{x}}_{k,v},u_{k},\sigma \right)={\left[\begin{array}{c} x^{+}\\ y^{+}\\ \theta^{+} \end{array}\right]}_{k+1}=\left[\begin{array}{c} {\hat{x}}_{k}+\left(\delta_{k,d}+\sigma_{d}\right)\cos\left({\hat{\theta }}_{k}\right)\\ {\hat{y}}_{k}+\left(\delta_{k,d}+\sigma_{d}\right)\sin\left({\hat{\theta }}_{k}\right)\\ {\hat{\theta }}_{k}+\delta_{k,\theta }+\sigma_{\theta } \end{array}\right]$$

where $u_{k}=\left[\begin{array}{c} \delta_{k,d}\\ \delta_{k,\theta } \end{array}\right]$ is the odometry output of the vehicle.

(A3) $$\hat{\Sigma }=\left[\begin{array}{cc} \sigma_{d} & 0\\ 0 & \sigma_{\theta } \end{array}\right]$$

The covariance is predicted with the following:

(A4) $${\hat{P}}_{k+1}^{+}=F_{x}{\hat{P}}_{k}{F_{x}}^{T}+F_{\sigma }\hat{\Sigma }F_{\sigma }^{T}$$

where $F_{x}$ and $F_{\sigma }$ are the Jacobians w.r.t. the state and the process noise σ, and are assumed to be linearly independent. Landmarks and the vehicle are considered independent [3]; therefore, Jacobians are composed block-wise:

(A5) $$F_{x}=\left[\begin{array}{ccc} F_{x_{v}} & \ldots & 0\\ \vdots & \ddots & \\ 0 & \; & F_{x_{lm}} \end{array}\right]$$

and

(A6) $$F_{\sigma }=\left[\begin{array}{ccc} F_{\sigma_{v}} & \ldots & 0\\ \vdots & \ddots & \\ 0 & \; & F_{\sigma_{lm}} \end{array}\right]$$

The vehicle Jacobians, $F_{x_{v}}$ and $F_{\sigma_{v}}$, are the state prediction functions evaluated at σ=0.

(A7) $$F_{x_{v}}=\left[\begin{array}{ccc} 1 & 0 & -\delta_{d}\;\sin\left({\hat{\theta }}_{k,v}\right)\\ 0 & 1 & \delta_{d}\;\cos\left({\hat{\theta }}_{k,v}\right)\\ 0 & 0 & 1 \end{array}\right]$$

and

(A8) $$F_{\sigma_{v}}=\left[\begin{array}{cc} \cos({\hat{\theta }}_{k,v}) & 0\\ \sin({\hat{\theta }}_{k,v}) & 0\\ 0 & 1 \end{array}\right]$$

Equation (3) yields the Jacobians for the static landmark states, as follows:

(A9) $$F_{x_{s}}=I_{2\times 2}$$

and

(A10) $$F_{\sigma_{s}}=0_{2\times 2}$$

which, by inspecting (4), reveals that the predicted uncertainty is equal to the updated uncertainty, as the identity matrix multiplication results in ${\hat{P}}_{k+1}^{+}={\hat{P}}_{k}$ for static landmarks. $F_{\sigma_{s}}=0_{2\times 2}$ ensures that no uncertainty is added through the process model in the second term of (4), regardless of model noise Σ.

### Appendix A.2. Update Step

The sensor functions are derived from Corke [7] and readers can refer to the textbook and associated codes (https://github.com/petercorke/robotics-toolbox-python (accessed on 1 September 2024) and [7] p. 220) for further details. The sensor used in this research is modeled as a range and bearing sensor, employing a nonlinear measurement function. For brevity, the subscripted *k* is dropped:

(A11) $$\begin{array}{c} z=h\left(x_{v},x_{lm}\right)=\left[\begin{array}{c} r\\ \beta \end{array}\right]\\ =\left[\begin{array}{c} \sqrt{{\left(y_{lm}-y_{v}\right)}^{2}+{\left(x_{lm}-x_{v}\right)}^{2}}\\ tan^{-1}\left(\frac{y_{lm}-y_{v}}{x_{lm}-x_{v}}-\theta_{v}\right) \end{array}\right]+\left[\begin{array}{c} \omega_{r}\\ \omega_{\beta } \end{array}\right] \end{array}$$

where *x* and *y* indicate the two dimensions of the plane and are the first two indices of each landmark in the corresponding state vector x. Measurements are incorporated with the innovation, which is determined by subtracting the expected measurements from the real measurements.

(A12) $$\nu =z_{k+1}-h\left(x_{k+1,v}^{+}\;,x_{k+1,lm}^{+}\right)$$

The sensor noise model used as part of the Kalman gain Equation (9) is as follows:

(A13) $$\hat{\Omega }=\left[\begin{array}{cc} \omega_{r} & 0\\ 0 & \omega_{\beta } \end{array}\right]$$

The standard Jacobians of (A11) w.r.t. $x_{v}$ and $x_{lm}$ are as follows:

(A14) $$H_{v}=\left[\begin{array}{ccc} -\frac{x_{lm}-x_{v}}{r} & -\frac{y_{lm}-y_{v}}{r} & 0\\ \frac{y_{lm}-y_{v}}{r^{2}} & -\frac{x_{lm}-x_{v}}{r^{2}} & -1 \end{array}\right]$$

and

(A15) $$H_{lm}=\left[\begin{array}{cc} \frac{x_{lm}-x_{v}}{r} & \frac{y_{lm}-y_{v}}{r}\\ -\frac{y_{lm}-y_{v}}{r^{2}} & \frac{x_{lm}-x_{v}}{r^{2}} \end{array}\right]$$

where the first two columns of $H_{v}=-H_{lm}$.

## Appendix B. Motion Models

Three models are used to predict the motion of the unknown dynamic landmark. A constant position linear model is also referred to as a static model. A constant velocity linear model is also referred to as a kinematic model. A nonlinear constant velocity model is also referred to as a body frame model.

### Appendix B.1. Constant Position Linear Model

The first assumes a constant position with changes through additive uncorrelated noise.

(A16) $$f_{lm}\left({\hat{x}}_{k,lm}\right)={\left[\begin{array}{c} x^{+}\\ y^{+} \end{array}\right]}_{k+1}={\left[\begin{array}{c} \hat{x}\\ \hat{y} \end{array}\right]}_{k}+\left[\begin{array}{c} \sigma_{x}\\ \sigma_{y} \end{array}\right]$$

where

- $x^{+}$ and $y^{+}$ are the predicted states for the next time step.
- ${\hat{x}}_{k}$ and ${\hat{y}}_{k}$ are the current state estimates.
- $\sigma_{x}$ and $\sigma_{y}$ are the process noise terms for the *x* and *y* coordinates.

The Jacobian $F_{x}$ remains the same as in the static model, $F_{x}=I_{2\times 2}$. Only $F_{\sigma }$ changes from a zero matrix to an identity matrix, resulting in increased uncertainty through the second term of (4), as follows:

$$F_{\sigma }=I_{2\times 2}$$

### Appendix B.2. Constant Velocity Linear Model

(A17) $$f_{lm}\left(x_{lm_{k}}\right)={\left[\begin{array}{c} x^{+}\\ y^{+}\\ {\dot{x}}^{+}\\ {\dot{y}}^{+} \end{array}\right]}_{k+1}=\left[\begin{array}{cccc} 1 & 0 & \Delta t & 0\\ 0 & 1 & 0 & \Delta t\\ 0 & 0 & 1 & 0\\ 0 & 0 & 0 & 1 \end{array}\right]{\left[\begin{array}{c} \hat{x}\\ \hat{y}\\ \hat{\dot{x}}\\ \hat{\dot{y}} \end{array}\right]}_{k}+\left[\begin{array}{cc} \Delta t & 0\\ 0 & \Delta t\\ 1 & 0\\ 0 & 1 \end{array}\right]\left[\begin{array}{c} \sigma_{\dot{x}}\\ \sigma_{\dot{y}} \end{array}\right]$$

where

- $x^{+}$, $y^{+}$, ${\dot{x}}^{+}$, and ${\dot{y}}^{+}$ are the predicted state variables (position and velocity) of the next time step.
- $\hat{x}$, $\hat{y}$, $\hat{\dot{x}}$, and $\hat{\dot{y}}$ are the estimated state variables at the current time step.
- Δt is the time difference between *k* and k+1.
- $\sigma_{\dot{x}}$ and $\sigma_{\dot{y}}$ are the process noise terms for the velocity components. There is no noise for the position; all motion is assumed to occur through velocity.

The Jacobians $F_{x}$ and $F_{\sigma }$ are defined as follows:

(A18) $$F_{x}=\left[\begin{array}{cccc} 1 & 0 & \Delta t & 0\\ 0 & 1 & 0 & \Delta t\\ 0 & 0 & 1 & 0\\ 0 & 0 & 0 & 1 \end{array}\right]$$

(A19) $$F_{\sigma }=\left[\begin{array}{cc} \Delta t & 0\\ 0 & \Delta t\\ 1 & 0\\ 0 & 1 \end{array}\right]$$

### Appendix B.3. Constant Velocity Nonlinear Model

One nonlinear motion model is used for moving landmarks. This model is closely related to the bicycle model used to simulate the original robot:

(A20) $$f_{lm}\left({\hat{x}}_{k,lm}\right)={\left[\begin{array}{c} x^{+}\\ y^{+}\\ v^{+}\\ \theta^{+} \end{array}\right]}_{k+1}=\left[\begin{array}{c} \hat{x}+\Delta t\;\left(\hat{v}+\sigma_{v}\right)\;\cos\left(\hat{\theta }+\sigma_{\theta }\right)\\ \hat{y}+\Delta t\;\left(\hat{v}+\sigma_{v}\right)\;\sin\left(\hat{\theta }+\sigma_{\theta }\right)\\ \hat{v}+\sigma_{v}\\ \hat{\theta }+\sigma_{\theta } \end{array}\right]$$

The Jacobians $F_{x}$ and $F_{v}$ are defined as follows:

(A21) $$F_{x}=\left[\begin{array}{cccc} 1 & 0 & \Delta t\;\cos(\hat{\theta }+\sigma_{\theta }) & -\Delta t\;\left(\hat{v}+\sigma_{v}\right)\;\sin\left(\hat{\theta }+\sigma_{\theta }\right)\\ 0 & 1 & \Delta t\;\sin(\hat{\theta }+\sigma_{\theta }) & \Delta t\;\left(\hat{v}+\sigma_{v}\right)\;\cos\left(\hat{\theta }+\sigma_{\theta }\right)\\ 0 & 0 & 1 & 0\\ 0 & 0 & 0 & 1 \end{array}\right]$$

(A22) $$F_{v}=\left[\begin{array}{cc} \Delta t\;\cos(\hat{\theta }+\sigma_{\theta }) & -\Delta t\;\left(\hat{v}+\sigma_{v}\right)\;\sin\left(\hat{\theta }+\sigma_{\theta }\right)\\ \Delta t\;\sin(\hat{\theta }+\sigma_{\theta }) & \Delta t\;\left(\hat{v}+\sigma_{v}\right)\;\cos\left(\hat{\theta }+\sigma_{\theta }\right)\\ 1 & 0\\ 0 & 1 \end{array}\right]$$

## Appendix C. Observation Models

A range and bearing sensor is used; therefore, the observation function (A11) does not change. The vehicle Jacobian $H_{v}$ and noise Jacobian $H_{w}$ are also unchanged compared to a static landmark model. Note that $H_{v}$ (A14) depends on the landmark states and, therefore, changes per landmark. A zero matrix is column-appended to $H_{lm}$ to complete the unobserved states, e.g., for the constant velocity motion models as presented in Appendix B.2 and Appendix B.3, as follows:

(A23) $$H_{lm}=\left[\begin{array}{cc} H_{lm} & \begin{array}{cc} 0 & 0\\ 0 & 0 \end{array} \end{array}\right]$$

This matrix is then inserted at the position of the corresponding landmark index of the state vector to form the final observation Jacobian, as follows:

(A24) $$H_{x}=\left[\begin{array}{ccccc} H_{v,i} & \ldots & H_{lm,i} & 0 & \ldots \\ H_{v,j} & \ldots & 0 & H_{lm,j} & \ldots \end{array}\right]$$

where the subscripts *i* and *j* indicate the Jacobians w.r.t to the landmarks *i* and *j*. (Please see Fenwick [6] and Equation (2.26) for further details).

Previously unseen landmarks are inserted in the following manner:

(A25) $${\hat{x}}_{k+1}=y\left({\hat{x}}_{k+1},z_{k}\right)=\left[\begin{array}{c} {\hat{x}}_{k+1}\\ g({\hat{x}}_{k+1},z_{k}) \end{array}\right]$$

(A26) $${\hat{P}}_{k+1}=Y_{z}\left(\begin{array}{cc} {\hat{P}}_{k+1} & 0\\ 0 & \hat{W} \end{array}\right)Y_{z}^{T}$$

With Jacobian:

(A27) $$Y_{z}=\left(\begin{array}{ccc} \; & I & \\ G_{x} & \; & 0 \end{array}\left|\begin{array}{c} 0\\ G_{z} \end{array}\right.\right)$$

The insertion function inserts the global states of a new landmark. The following is an example of a linear kinematic motion model described in Appendix B.2:

(A28) $$g\left({\hat{x}}_{k+1},z_{k}\right)\left(\begin{array}{c} {\hat{x}}_{v}+r\cos\left({\hat{\theta }}_{v}+\beta \right)\\ {\hat{y}}_{v}+r\sin\left({\hat{\theta }}_{v}+\beta \right)\\ {\dot{x}}_{max}\\ {\dot{y}}_{max} \end{array}\right)$$

We have the following Jacobians:

(A29) $$G_{x}=\left[\begin{array}{ccc} 1 & 0 & -r\sin({\hat{\theta }}_{v}+\beta )\\ 0 & 1 & r\cos({\hat{\theta }}_{v}+\beta )\\ 0 & 0 & 0\\ 0 & 0 & 0 \end{array}\right]$$

(A30) $$G_{z}=\left[\begin{array}{cc} \cos({\hat{\theta }}_{v}+\beta ) & -r\sin({\hat{\theta }}_{v}+\beta )\\ \sin({\hat{\theta }}_{v}+\beta ) & r\cos({\hat{\theta }}_{v}+\beta )\\ 0 & 0\\ 0 & 0 \end{array}\right]$$

The generalized insertion equation for multiple landmarks is generated analogous to update Equation (A24), with a prepended identity matrix, $I_{M\times M}$, and *j* representing the last new landmark, as follows:

(A31) $$Y_{z}=\left(\begin{array}{ccc} \; & I_{M\times M} & \\ G_{x_{1}} & \; & 0\\ G_{x_{2}} & \; & 0\\ \vdots & \; & \vdots \\ G_{x_{j}} & \; & 0 \end{array}\left|\begin{array}{cccc} 0 & 0 & \ldots & 0\\ G_{z_{1}} & 0 & \ldots & 0\\ 0 & G_{z_{2}} & \ldots & 0\\ \vdots & \ddots & \vdots \\ 0 & 0 & \ldots & G_{z_{j}} \end{array}\right.\right)$$

where subscripts need to correspond to the Jacobians for each landmark model, 0 matrices are filled according to empty spaces, and *M* is the dimension of the state vector, the map dimension, at time *k*.

## Appendix D. Occlusions

Figure A1 shows the results of reducing the sensing range to 10, which excludes landmarks from the observation. While ATE, rotation, and translation distance exhibit behavior similar to full observability, as shown in Figure 6, dynamic ATE is reduced for kinematic models. This effect becomes more pronounced while further reducing the sensing range, as displayed in Figure 9:
